# Isolated Encephalopathy Without Severe Disease in a COVID-19 Patient: Case Presentation and Workup Strategies

**DOI:** 10.7759/cureus.13277

**Published:** 2021-02-11

**Authors:** Lindsay S McAlpine, Mary Barden, Adeel S Zubair, Sirisha Sanamandra

**Affiliations:** 1 Neurology, Yale School of Medicine, New Haven, USA

**Keywords:** encephalopathy, confusion, covid-19, coronavirus

## Abstract

Severe acute respiratory syndrome coronavirus 2 (SARS-CoV-2) is the virus responsible for the human coronavirus disease 2019 (COVID-19). It has been shown to cause not only respiratory disease but has manifestation in multiple organ systems. Encephalopathy has been shown to be associated with COVID-19; typically, it presents with other findings of the disease including headache, respiratory dysfunction, and fevers. We report a case of a 60-year-old man with hypertension who presented with confusion and cognitive decline concerning for encephalopathy and was found to have COVID-19. On neurologic examination, he had impaired episodic memory, attention, and comprehension with intact motor and sensory examination. Other than fatigue, the patient had no other common COVID-19 symptoms.

## Introduction

Severe acute respiratory syndrome coronavirus 2 (SARS-CoV-2) emerged in December of 2019 and caused human coronavirus disease 2019 (COVID-19) which was initially identified as causing a severe respiratory disease [1-2]. With more patients contracting the virus and becoming infected, it was noted that the disease can affect a variety of organ systems. Neurological complications have been reported with common symptoms being headache, anosmia, ageusia, weakness, and encephalopathy [1-2]. The majority of patients presenting with these symptoms also have other manifestations of the disease [1-2]. We present a case of a 60-year-old man presenting with isolated encephalopathy as a manifestation of COVID-19 and discuss the workup for approaching patients with encephalopathy. 

## Case presentation

A 60-year-old man with hypertension presented with two weeks of cognitive decline with no respiratory symptoms. At baseline, he was independent in activities of daily living, handled his own finances, and worked full-time as a custodian. He described feeling “off” and unmotivated but otherwise had no specific complaints. His cognitive decline became so noticeable that his coworkers advised him to stay home. He denied loss of consciousness, focal weakness, dizziness, abnormal movements, or headache. He also denied recreational drug use or toxic exposures. He was a former tobacco smoker and had a history of alcohol use disorder with his last drink being four months prior to presentation.

**Figure 1:**
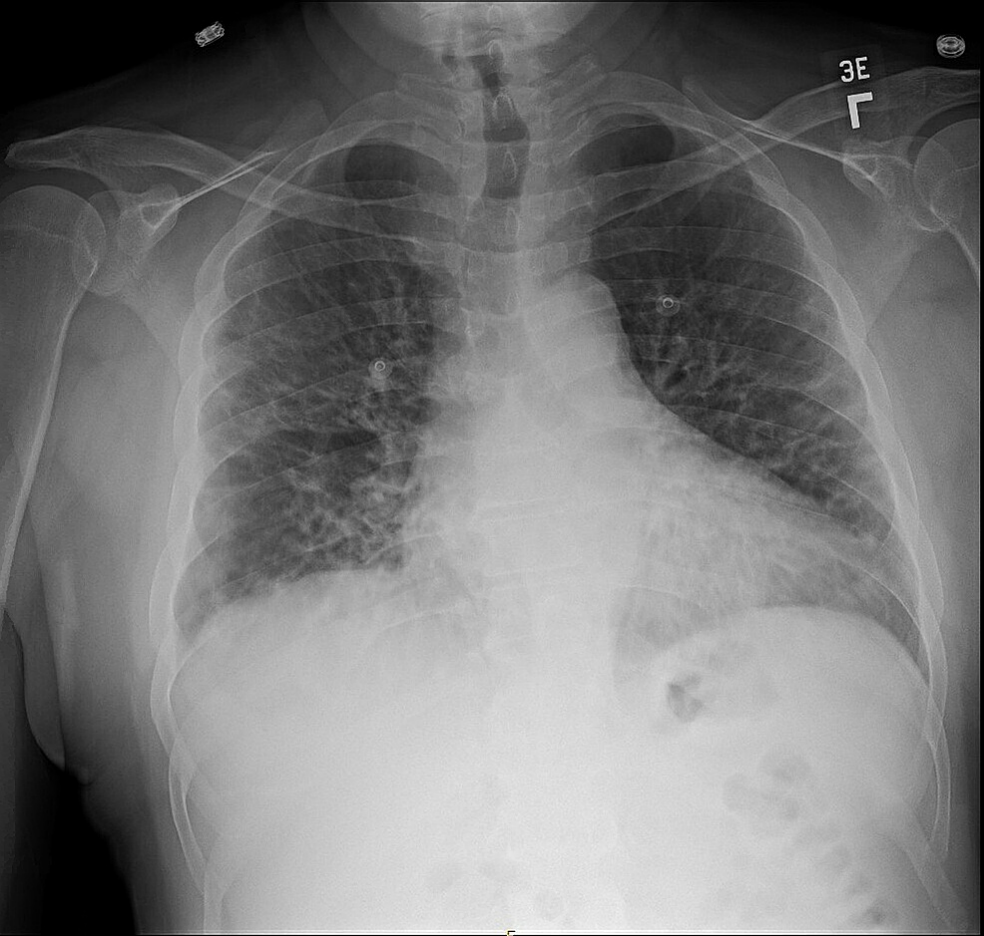
Chest X-ray at time of presentation Chest X-ray showing a normal cardiomediastinal silhouette with evidence of bilateral peripheral hazy opacities. No pleural effusion or pneumothorax is seen.

While in the emergency department, his temperature was 101.2 Fahrenheit, pulse 115 beats per minute, blood pressure 126/83 mmHg, and oxygen saturation was 92% on room air. SARS-CoV-2 reverse transcription‐polymerase chain reaction (RT‐PCR) was positive. On admission, he met the systemic inflammatory response syndrome (SIRS) criteria without shock based on fever, heart rate, and white blood cell (WBC) count [3]. On neurologic examination, he was disoriented to numerical date. His speech was fluent without paraphasic errors but notable for significant bradyphrenia. Naming and repetition were intact; however, comprehension of complex commands, simple math, and attention were all significantly impaired. He gave vague and variable accounts of recent events, which pointed to difficulty with short-term memory. Cranial nerve, motor, sensory, reflex, and gait examinations were otherwise normal. There was no nuchal rigidity. Laboratory studies revealed an elevated creatinine of 1.46 mg/dL (ref: 0.4 - 1.3 mg/dL), WBC count 13.0 μL (4.0 - 10.0 x1000 μL), alanine aminotransferase 154 U/L (ref: 6 -34 U/L), and aspartate amino transferase 190 U/L (ref: 11 - 33). His sepsis-related organ failure assessment (SOFA) score was 2 based on creatinine and confusion. A chest X-ray demonstrated bilateral hazy opacities (Figure 1). Inflammatory markers were also significantly elevated, including C-reactive protein (CRP) 156.5 mg/L (ref: < 1.0 mg/L), ferritin 3,853 ng/mL (ref: 30 -400 ng/mL), D-dimer 0.85 mg/L (ref: < 0.6 mg/L), interleukin-6 4.6 pg/mL (ref: <2.0 pg/mL), and interleukin-2 receptor 1,734.7 pg/mL (ref: 175.3 - 858.2 pg/mL) (Table 1).

**Table 1:** Laboratory values upon presentation SARS-CoV-2: severe acute respiratory syndrome coronavirus 2; RT-PCR: reverse transcription polymerase chain reaction

	Value
SARS-CoV-2 RT-PCR	Positive
Creatinine	1.46 mg/dL (ref. 0.4 – 1.3 mg/dL)
White Blood Cell Count	13.0 μL (4.0 – 10.0 x1000 μL)
Alanine aminotransferase	154 U/L (ref 6 –34 U/L)
Aspartate amino transferase	190 U/L (ref 11 – 33)
C-reactive protein (CRP)	156.5 mg/L (ref. < 1.0 mg/L)
Ferritin	3,853 ng/mL (ref. 30 -400 ng/mL)
D-dimer	0.85 mg/L (ref. < 0.6 mg/L)
interleukin-6	4.6 pg/mL (ref. <2.0 pg/mL)
interleukin-2 receptor	1,734.7 pg/mL (ref. 175.3 – 858.2 pg/mL)

To rule out treatable causes of encephalopathy, the patient underwent a thorough work-up. Laboratory tests for metabolic disturbances, toxicology, co-morbid infection, and reversible causes of dementia were overall normal. He had a normal sodium of 139 mmol/L (136 - 144 mmol/L), glucose 107 mg/dL (70 - 100 mg/dL), vitamin B12 1,251 pg/mL (232 - 1,245 pg/mL), and mildly elevated blood urea nitrogen of 39 mg/dL (4 - 19 mg/dL). Vitamin B1 was 9 nmol/L (8 - 30 nmol/L) and ammonia was low at <10 umol/L (11 - 35 umol/L). His mild transaminitis resolved early in his admission. Despite an acute kidney injury (baseline creatinine within normal limits), there were no marked metabolic abnormalities to explain his indolent presentation. Toxicology of the urine and serum were negative and treponema pallidum antibodies were negative in serum.

**Figure 2:**
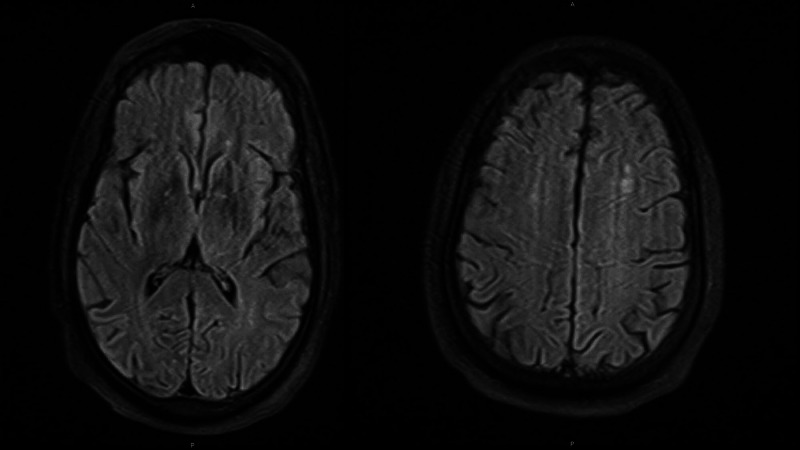
MRI of the brain, without gadolinium contrast (fluid attenuated inversion recovery (FLAIR) sequence) There is no acute intracranial hemorrhage or acute infarct with no evidence of mass effect or midline shift. Several scattered foci of FLAIR signal are noted in the periventricular and subcortical white matter, likely sequela of small vessel ischemic disease.

**Figure 3:**
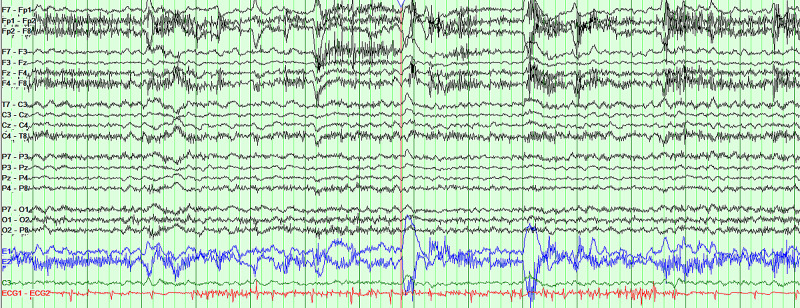
Electroencephalography (EEG) Mild to moderate generalized slowing, suggesting diffuse or multifocal dysfunction. No seizures or epileptiform patterns seen.

Analysis of the cerebrospinal fluid (CSF) revealed 0 nucleated cells, glucose of 60 mg/dL, and protein of 32.5 mg/dL (normal less than 45 mg/dL), demonstrating no evidence of inflammation in the CNS. Additional studies were negative, including a viral panel and bacterial culture. Given the concern for a possible post-infectious autoimmune-mediated encephalitis, a Mayo Laboratory autoimmune encephalopathy panel was also sent on the CSF and was negative. Next-of-kin did not consent to CSF SARS-CoV-2 PCR, antibody, and cytokine testing for the patient. An MRI of the Brain without gadolinium contrast demonstrated scattered foci of fluid attenuated inversion recovery (FLAIR) hyperintensity in the periventricular and subcortical white matter (Figure 2). These were likely sequelae of chronic small vessel disease. There was no structural cause for encephalopathy identified and an EEG was performed to assess for non-convulsive status epilepticus. An extended six-hour EEG demonstrated mild to moderate generalized slowing without seizures or epileptiform patterns (Figure 3). The patient had an awake background rhythm with theta and a 6-7 Hz posterior dominant rhythm. Otherwise, the background and sleep rhythms were normal. The findings suggested diffuse, multifocal dysfunction.

Upon admission, he received a five-day course of remdesivir to treat COVID-19. He was also provided supplemental thiamine and supportive care. Of note, no steroids were given. His cognitive symptoms improved over the week of his admission. Inflammatory markers at the end of admission had improved significantly. His CRP was 27.4 mg/L (ref: < 1.0 mg/L) and ferritin 1,358 ng/mL (ref: 30 -400 ng/mL). He was seen in follow-up telehealth visit three weeks after discharge and was found to have good attention on letter string task and digit span. He reported feeling back to normal with improvement noted in laboratory values.

## Discussion

Early in the pandemic, encephalopathy was identified as a common neurological complication of COVID-19 [1-2]. Encephalopathy typically develops in COVID-19 patients with sepsis and multiorgan dysfunction syndrome (MODS) but rarely it can be the presenting symptom of COVID-19. In critically ill COVID-19 patients, it is difficult to distinguish encephalopathy due to hypoxia, sepsis, MODS, or metabolic dysfunction from primary neurological disease. Toxic metabolic encephalopathy can be seen in the setting of hypoglycemia, hepatic dysfunction, uremia, hypo- or hypernatremia, electrolyte imbalances, endocrinopathies, or sepsis alone. Septic associated encephalopathy (SAE) is a well-described phenomena not attributable to CNS infection but rather to the systemic response to infection. Inflammation, oxidative stress, altered blood-brain barrier permeability, endothelial injury, altered cerebral perfusion, and bacterial endotoxins all likely contribute to SAE [4].

Patients may also experience encephalopathy due to central nervous system pathology including encephalitis, seizures, ischemic or hemorrhagic stroke, posterior reversible encephalopathy syndrome (PRES), hypoxic-ischemic insult, and mass lesions. The patient here presented with only a global encephalopathy and otherwise normal exam without typical COVID-19 symptoms such as cough, chills, dyspnea, myalgias, headache, anosmia, or diarrhea. His encephalopathy was out of proportion to his otherwise asymptomatic COVID-19 illness and mild metabolic abnormalities including a mild acute kidney injury and transaminitis [5]. However, his markedly elevated CRP and IL-2r demonstrated significant systemic inflammation and were more consistent with critically ill patients [6].

The mechanism of encephalopathy has yet to be determined but early studies have suggested that inflammation, increased permeability of the blood brain barrier, and direct viral invasion may play a role [2]. Patients with COVID-19 encephalopathy have been shown to have elevated protein and SARS-CoV-2 antibodies in their CSF [7]. Whether the antibodies are present in response to virus in the CSF or due to crossing the blood brain barriers is unclear. Patients with neurological complications of COVID-19 have also been found to have significant inflammation in the CSF, including elevated interleukin-6 (IL-6), IL-8, IL-10, interferon-gamma induced protein (IP-10), tumor necrosis factor- α (TNF-α), and monocyte chemoattractant protein-1 (MCP-1).7 Other findings demonstrated in critically ill patients that could contribute to encephalopathy include bilateral frontotemporal hypoperfusion, leptomeningeal enhancement, and hypoxic changes on pathology [8,9].

There are several neurological complications of COVID-19 that can mimic encephalopathy. We would not expect an otherwise healthy 60-year-old man to develop such a significant encephalopathy. In addition to typical toxic-metabolic etiologies, care teams should consider thoroughly evaluating for underlying organic lesions with imaging, CSF studies, and EEG. There have been several cases of encephalitis due to COVID-19 reported, but overall it remains a rare occurrence [2,10]. One case of encephalitis appeared to be steroid-responsive, suggesting an underlying autoimmune pathophysiology, rather than direct viral invasion [11]. There have been several cases of suspected acute disseminated encephalomyelitis (ADEM), which further supports an autoimmune-mediated process underlying COVID-19 damage to the CNS [12,13]. Brain pathology in one patient showed axonal injury, perivascular inflammation, and macrophage infiltration consistent with ADEM [14]. Lastly, a handful of acute necrotizing encephalopathy (ANE) cases have been reported [15]. ANE typically develops in the setting of severe viral infections and systemic inflammation.

It is interesting to note that this patient developed encephalopathy as a presenting symptom without developing a critical illness or respiratory failure. On EEG, he was found to have diffuse slowing, which is a common finding in COVID-19 encephalopathy. A study of 13 patients show background slowing to 4-8 Hz with theta and delta predominately [16]. Notably, no epileptiform abnormalities were seen. In this study, three patients showed resolution of EEG findings with resolution of encephalopathy [16]. It is likely that the cytokine storm in response to COVID-19 contributes significantly to immune dysregulation and encephalopathy. Future pathological, clinical, imaging, and serologic studies may further elucidate the underlying mechanism of COVID-19 encephalopathy.

## Conclusions

Encephalopathy can present as a result of a variety of different conditions, including COVID-19. While it typically presents in the setting of other aspects of the disease, it can rarely be found as the solitary clinical symptom. It is important have a broad differential diagnosis when evaluating these patients and a thorough history coupled with clinical examination and diagnostic testing can rule out other potential causes.
